# Resource Allocation and 3D Deployment of UAVs-Assisted MEC Network with Air-Ground Cooperation

**DOI:** 10.3390/s22072590

**Published:** 2022-03-28

**Authors:** Jinming Huang, Sijie Xu, Jun Zhang, Yi Wu

**Affiliations:** 1 Fujian Provincial Engineering Technology Research Center of Photoelectric Sensing Application, Fujian Normal University, Fuzhou 350007, China; qsx20200796@student.fjnu.edu.cn (J.H.); qsx20210835@student.fjnu.edu.cn (S.X.); 2 Jiangsu Key Laboratory of Wireless Communications, Nanjing University of Posts and Telecommunications, Nanjing 210003, China; zhangjun@njupt.edu.cn

**Keywords:** mobile edge computing, UAV communication, resource allocation, 3D deployment, air-ground cooperation

## Abstract

Equipping an unmanned aerial vehicle (UAV) with a mobile edge computing (MEC) server is an interesting technique for assisting terminal devices (TDs) to complete their delay sensitive computing tasks. In this paper, we investigate a UAV-assisted MEC network with air–ground cooperation, where both UAV and ground access point (GAP) have a direct link with TDs and undertake computing tasks cooperatively. We set out to minimize the maximum delay among TDs by optimizing the resource allocation of the system and by three-dimensional (3D) deployment of UAVs. Specifically, we propose an iterative algorithm by jointly optimizing UAV–TD association, UAV horizontal location, UAV vertical location, bandwidth allocation, and task split ratio. However, the overall optimization problem will be a mixed-integer nonlinear programming (MINLP) problem, which is hard to deal with. Thus, we adopt successive convex approximation (SCA) and block coordinate descent (BCD) methods to obtain a solution. The simulation results have shown that our proposed algorithm is efficient and has a great performance compared to other benchmark schemes.

## 1. Introduction

In recent years, with the development of the Internet of Things (IoT) and small mobile devices, more and more applications are employed in daily life, such as face recognition, automatic navigation, video processing, and unmanned driving [1,2,3,4]. On the one hand, these applications are significantly improving our quality of experience (QoE). On the other hand, they are usually computation intensive and delay sensitive, which presents a great challenge for the independent terminal device (TD) in dealing with large amounts of data in a short time with limited computing capability and battery energy [5,6,7]. To solve this problem, a promising mobile edge computing (MEC) solution has been proposed which services terminal devices such as cloud computing with its ample computing resources. Unlike traditional cloud computing, the MEC server overcomes the difficulty presented by the fact of cloud computing servers being distant from the TDs, by being located at the infrastructure-based edge of wireless networks in proximity to TDs [8]. By offloading some or all computation-intensive tasks to the MEC server, the quality of computation can be significantly improved.

**Figure 1:**
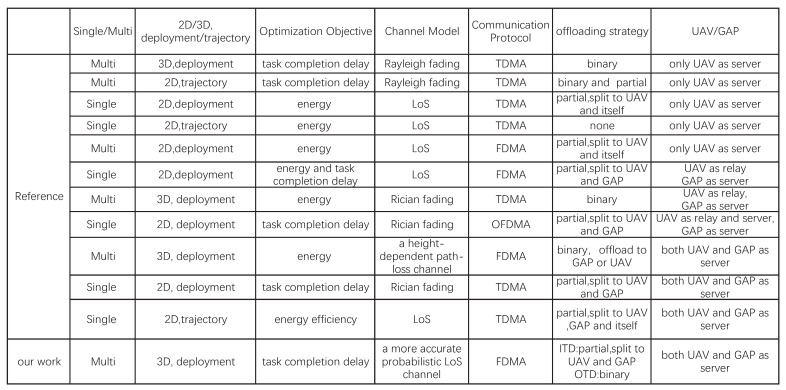
Related works on UAV-assisted MEC systems. The references are in order from top to bottom: [9,10,11,12,13,14,15,16,17,18,19].

There has been much research on the already mature traditional MEC system. However, MEC systems with a ground access point (GAP) have some limitations. Firstly, with the number of TDs increasing dramatically, the density of TDs is more concentrated, which may cause blocks in the offloading task and overload the MEC server. Secondly, each ground access point has a certain coverage in complex environments defined by the obstructions of tall buildings and trees, so some TDs will be located outside of coverage and unable to receive MEC service. Thirdly, the fixed position of GAPs is unsuitable given the mobility of TDs. These prominent weaknesses of a traditional ground access point force researchers to seek a useful tool to improve the computing capacity of MEC systems, such as unmanned aerial vehicles (UAVs) (See Figure 1).

As a flexible air platform with high mobility, UAVs provide a better communication link for users and are widely used in wireless communication systems, disaster detection, and agriculture investigation. Furthermore, a UAV equipped with a computing server can be applied in a MEC system as an air access point. Compared with traditional ground access points, it can greatly shorten the offloading time and be deployed more easily. In [9], the authors minimized the total time required for the UAVs to complete the computing tasks of users without calculation capability by joint association of TDs and UAVs and 3D deployment of UAVs. Also focused on the delay topic, the authors in [10] aimed to minimize task completion time by scheduling, computation resource allocation, and UAVs’ trajectories, with discussion of two offloading strategies. The authors in [11] studied the weighted-sum energy consumption problem in a UAV-assisted MEC system by joint task offloading and local computing design. In [12], the authors minimized the total energy consumption of the NOMA-based MEC networks underlaying the UAV with time, computation capacity, and UAV trajectory. The work in [13] aimed to minimize the energy consumption of terminal devices and UAVs by joint optimizing device association, task assignment and computing resource allocation.

The aforementioned research [9,10,11,12,13] investigating MEC systems only considered UAV servers. It is notable that UAVs have better offloading links but are poor in computing with limited computing resources compared to GAPs. Thus, to further improve the performance of MEC systems, more and more papers have investigated the cooperation between GAPs and UAVs. One cooperation scenario features a UAV acting as relay to deliver the computing task from TDs to GAP. The authors in [14] minimized a cost function around both energy consumption and system delay in a ground-based Internet of Things (IoT) system, using a UAV as relay. The authors in [15] investigated a UAV-relaying-assisted MEC system to minimize the weighted sum of transmission and hovering energy consumptions. In [16], the authors aimed to minimize the task completion delay in a UAV-relaying-assisted MEC system, where the UAV not only acted as a MEC server to complete part of a computing task, but also as relay to deliver the rest of the task to a GAP for execution.

Meanwhile, it is noted that some TDs located close to a GAP can also offload tasks effectively without a UAV relay. Thus, there is another scenario for air–ground cooperation, i.e., both GAP and UAV directly linked to TDs independently, with GAPs mainly servicing the TDs near it and UAVs servicing the TDs far from the GAP. In [17], subject to binary offloading strategy, the authors minimized the total energy consumption of UEs by jointly optimizing uplink power control, channel allocation, and computation capacity allocation. With a more effective partial offloading strategy, the authors in [18] aimed to minimize the maximum delay of TDs by designing a cooperative sky-ground mobile edge computing system, which has a significantly better performance compared to an only UAV-assisted MEC system and ground-only MEC system. In [19], the authors proposed a weighted computation efficiency maximization framework for MEC systems by jointly optimizing the computation resource scheduling, bandwidth allocation, and UAV’s trajectory, where the benefits of the proposal are more prominent when handling computation intensive latency-critical tasks. However, [18,19] are only considered the case of one UAV and investigate the condition that each time slot only services one TD with TDMA. Furthermore, the fixed height restriction of the UAV limited its excellent performance, and simple channel condition assumptions made its results inaccurate.

Motivated by previous works, in this paper, based on the ground MEC system, we introduce UAVs as air access point to form a novel UAV-assisted MEC system with air–ground cooperation, where UAVs and GAPs undertake computing task cooperatively. Specifically, we aim to consistently minimize the maximum delay among TDs. To this end, we proposed an iterative algorithm by jointly optimizing UAV–TD association, UAV horizontal location, UAV vertical location, bandwidth allocation, and task split ratio. Several advantages can be obtained by such design. First, with the introduction and deployment design of UAVs, the service coverage and quality of a MEC system is greatly expanded. Second, by resource allocation such as the association between TDs and UAVs, task split radio and bandwidth allocation, a tradeoff of delay among different TDs is achieved.

The major contributions of the paper are shown as follows:

- To the best of our knowledge, our paper is the first to propose the novel framework of a UAV-assisted MEC system with air–ground cooperation, where UAVs and GAPs undertake computing tasks cooperatively. For further practicality, we discard the usual simplified line-of-sight (LoS) links and adopt a more accurate probabilistic channel [20], which is obtained by simulation and data regression methods.
- Our goal is to minimize the maximum delay among TDs. To this end, we propose an iterative algorithm by jointly optimizing UAV–TD association, UAV horizontal location, UAV vertical location, bandwidth allocation, and task split radio.
- To solve this mixed-integer nonlinear programming (MINLP), we adopt successive convex approximation (SCA) and block coordinate descent (BCD) method. Meanwhile, the coverage and complexity of the algorithm has been analysed. Besides, simulations are conducted to test the efficiency of our proposed algorithm and to validate its better performance compared to other benchmark algorithms.

The rest of this paper is organized as follows: The system model and the problem formulation for a UAV-assisted MEC system are showed in Section 2. In Section 3, we propose an efficient iterative algorithm, divide the overall optimization problem into five subproblems, and then solve them alternately. Section 6 displays our simulation results to demonstrate the performance of our proposed design. Lastly, in Section 5, we conclude the paper.

## 2. System Model and Problem Formulation

### 2.1. MEC Network Model

**Figure 2:**
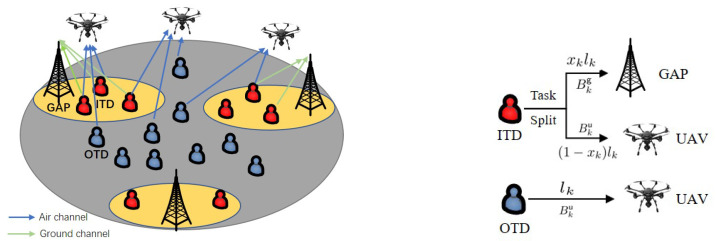
A UAVs-assisted MEC system with air-ground cooperation.

In this paper, we consider a newly UAVs-assisted MEC system with air-ground cooperation, where several GAPs and *M* UAVs with an index set $M\overset{\Delta }{\;=}\{{\mathrm{UAV}}_{1},{\mathrm{UAV}}_{2},\ldots ,{\mathrm{UAV}}_{M}\}$ offer computing service for *K* ground TDs $K\overset{\Delta }{\;=}\{{\mathrm{TD}}_{1},{\mathrm{TD}}_{2},\ldots ,{\mathrm{TD}}_{K}\}$ cooperatively, as shown in Figure 2. All the communication nodes (TD, GAP, and UAV) are equipped with single antennae. It is assumed that each GAP has a limited coverage without overlaps. Thus, we can divide all the TDs into two types with the locations of GAPs and TDs known in advance. One type is TD within the coverage of GAPs (ITD). The other type is TD outside the coverage of GAPs (OTD). The task of ITD can be divided into two parts with a task splitting ratio, where one part is offloaded to a GAP and the other part is offloaded to a UAV. The OTD only completes computing tasks by offloading them to a UAV. We set the number of ITD as *N*, where index is $N\overset{\Delta }{\;=}\left\{{\mathrm{TD}}_{1},{\mathrm{TD}}_{2},\ldots ,{\mathrm{TD}}_{N}\right\}\left(N\in K\right)$. Then, OTD is denoted by $\left\{{\mathrm{TD}}_{N+1},{\mathrm{TD}}_{N+2},\ldots ,{\mathrm{TD}}_{K}\right\}$.

### 2.2. Channel Model

Without loss of generality, we adopt the 3D cartesian coordinate system to denote the locations. The locations of TDs are represented by $(w_{k},0),k\in K$, where $w_{k}={\left[x_{k},y_{k}\right]}^{T}\in R^{2\times 1}$ denotes the horizontal location of TD${}_{k}$. Similarly, GAPs’ locations are represented by $\left(w_{g},H_{g}\right)$, where $w_{g}={\left[x_{g},y_{g}\right]}^{T}\in R^{2\times 1}$ and $H_{g}$ denotes the horizontal coordinate and height of the GAPs. UAVs’ locations are represented by $(q_{m},z_{m}),m\in M$, where $q_{m}={\left[x_{m},y_{m}\right]}^{T}\in R^{2\times 1}$ and $z_{m}$ denote the horizontal coordinate and vertical coordinate of UAV${}_{m}$, respectively.

Then the distance between TD${}_{k}$ and its associated GAP and between TD${}_{k}$ and UAV${}_{m}$ can be denoted as, respectively,

(1a)(1b)

There are two kinds of channels in our system. The channel from TD to GAP is called the ground channel and the channel from TD to UAV is called the air channel. It is worth noting that all TDs have an air channel but only ITDs have a ground channel.

For complex environments with obstructions in the form of tall buildings or trees, the ground channel is modeled using the Rayleigh fading model, where the channel power gain can be described as

(2) $$h_{k,g}=\beta_{0}d_{k,g}^{-a_{R}}\zeta_{k,g},$$

where $\beta_{0}$ denotes the reference channel power when the distance $d_{0}=1$ meter, $a_{R}$ denotes path loss exponent of Rayleigh channel, $\zeta_{k,g}$ denotes the Rayleigh fading coefficient subject to the exponential distribution with unit mean.

In this paper, to allow each TD to access computing service simultaneously and continuously, frequency division multiple access (FDMA) protocol is adopted in our system, where each transmission channel occupies a different frequency band without overlapped bandwidth to avoid the interference. Then, we can get the transmission rate (bit/s) from TD${}_{k}$ to its associated GAP of ground channel as

(3) $$r_{k}^{g}=B_{k}^{g}\log_{2}\left(1+\frac{ph_{k,g}}{N_{0}B_{k}^{g}}\right),$$

where $B_{k}^{g}$ denotes the ground channel bandwidth of TD${}_{k}$, $N_{0}$ denotes the noise power spectral density at UAV, *p* denotes the transmission power of TD${}_{k}$.

With high mobility of UAV, the air channel will appear two different states, LoS channel state and NLoS channel state, respectively. To describe the feature of channel more accurately, we introduce a probabilistic LoS channel model which obtained by simulation and data regression methods [20]. Then, the probability of LoS state between TD${}_{k}$ and UAV${}_{m}$ is specifically denoted by

(4) $$P_{k,m}^{\mathrm{LoS}}=C_{3}+\frac{C_{4}}{1+e^{-(C_{1}+C_{2}\theta_{k,m})}},$$

where $C_{3}<0$, $C_{4}>0$, $C_{1}>0$ are the constants about specific environment, and $C_{1}$ and $C_{2}$ are constants with $C_{1}+C_{2}=1$, $\theta_{k,m}$ denoting the elevation angle between TD${}_{k}$ and UAV${}_{m}$, i.g.,

(5) $$\theta_{k,m}=\frac{180}{\pi }\arctan\left(\frac{H_{m}}{∥q_{m}-w_{k}∥}\right).$$

Then the transmission rate of LoS state between TD${}_{k}$ and UAV${}_{m}$ is denoted by

(6) $$r_{k,m}^{\mathrm{LoS}}=B_{k}^{u}\log_{2}\left(1+\frac{ph_{k,m}^{\mathrm{LoS}}}{\Gamma N_{0}B_{k}^{u}}\right),$$

(7) $$h_{k,m}^{\mathrm{LoS}}=\beta_{0}d_{k,m}^{-a_{L}},$$

where $B_{k}^{u}$ is the air channel bandwidth of the TD${}_{k}$, Γ is the signal-to-noise ratio (SNR) gap between the practical coding method and the theoretical Gaussian signaling, $h_{k,m}^{\mathrm{LoS}}$ represents the channel power gain of LoS channel, $a_{L}$ represents path loss exponent of LoS channel.

In a practical scenario, the transmitting rate of the LoS state is much greater than the NLoS state $\left(r_{k,m}^{\mathrm{LoS}}\gg r_{k,m}^{\mathrm{NLoS}}\right)$ so that the NLoS state can be omitted [20]. Thus, the final average transmission rate (bit/s) of the air channel between TD${}_{k}$ and UAV${}_{m}$ can be approximately denoted by

(8) $$\begin{array}{cc} R_{k,m} & =P_{k,m}^{\mathrm{LoS}}r_{k,m}^{\mathrm{LoS}}+\left(1-P_{k,m}^{\mathrm{LoS}}\right)r_{k,m}^{\mathrm{NLoS}}\\ \; & \geq P_{k,m}^{\mathrm{LoS}}r_{k,m}^{\mathrm{LoS}}\\ \; & \overset{\Delta }{\;=}(C_{3}+\frac{C_{4}}{1+e^{-(C_{1}+C_{2}\theta_{k,m})}})B_{k}^{u}\log_{2}\left(1+\frac{ph_{k,m}^{\mathrm{LoS}}}{\Gamma N_{0}B_{k}^{u}}\right). \end{array}$$

### 2.3. System Delay Model

$L_{k}=\left\{l_{k},c_{k}\right\}$ is denoted as the computing task of TD${}_{k}$, where $l_{k}$ (bit) is the size of task data and $c_{k}$ (cycles/bit) is the number of CPU cycles required to deal with 1-bit of task data. Assuming adequate computing resource of the UAV and GAP, $f^{u}$ and $f^{g}$ represent the computing resource that UAV and GAP distribute to each computing task. Thus, we can get the computing delay of UAV and GAP as $t_{k}^{u}=\frac{l_{k}c_{k}}{f^{u}}$ and $t_{k}^{g}=\frac{l_{k}c_{k}}{f^{g}}$, respectively. It is assumed that the transmission process and computing process of each task cannot be done at the same time, i.e., the computing process will not begin until the transmitting process is completed. Furthermore, the computation result takes much less time than the task input, thus we omit the time for sending back of the result. Then, the task completion delay of each TD is composed of transmitting delay and computing delay.

Specifically, for the air channel, we suppose that each TD can only choose one UAV to offload the computing task at most, which is denoted by

(9) $$\begin{array}{cc} \; & \sum_{m=1}^{M}a_{k,m}\leq 1,\;\forall k, \end{array}$$

(10) $$\begin{array}{cc} \; & a_{k,m}\in \left\{0,1\right\},\;\forall k,m, \end{array}$$

where $a_{k,m}$ is a binary variable to denote the association between TDs and UAVs, which indicates that TD${}_{k}$ offload task to UAV${}_{m}$ if $a_{k,m}$ = 1; Otherwise, $a_{k,m}$ = 0. Then the delay of TD${}_{k}$ to complete the task by offloading it to UAV${}_{m}$ can be described as

(11) $$T_{k,m}=a_{k,m}\left(\frac{x_{k}l_{k}}{R_{k,m}}+x_{k}t_{k}^{u}\right),\;\forall k,m,$$

where $x_{k}$ denotes the task split ratio to UAV of TD${}_{k}$. Then, $\left(1-x_{k}\right)$ denotes the task split ratio to its associated GAP. Furthermore, $x_{k}=1$ represents that the entire task is offloaded to the UAV, conversely $x_{k}=0$ denotes that the entire task is offloaded to GAP.

For ground channel, because only ITD has a ground channel the delay can be described as

(12) $$T_{k}^{g}=\left\{\begin{array}{c} \left(\frac{\left(1-x_{k}\right)l_{k}}{r_{k}^{g}}+\left(1-x_{k}\right)t_{k}^{g}\right),\forall k\in N,\\ 0,\;\;\;\;\;\forall k\in K,k\notin N. \end{array}\right.$$

Finally, the total system delay is the maximum delay among TDs, which is represented by

(13) $$\max\{T_{k}^{g},T_{k,m},\forall k,m\}.$$

### 2.4. Problem Formulation

In this paper, with fairness among TDs, our goal is to minimize system delay by finding five optimal sets $A=\{a_{k,m},\forall k,m\}$, $Q=\{q_{m},\forall m\}$, $Z=\{z_{m},\forall m\}$, $B=\{B_{k}^{u},B_{k}^{g},\forall k\}$, $X=\{x_{k},\forall k\}$. Correspondingly, our optimization problem is formulated as follows

(P1):min{A,Q,Z,B,X}max{∀k,m}{Tkg,Tk,m},(14a)(14b)(14c)(14d)(15e)(14f)(14g)(14h)

where (14a) and (14b) are the constraints about the association between TDs and UAVs, (14c) is the constraint of the flying height of UAV, $H_{\min}$ denotes the minimum height and $H_{\max}$ denotes the maximum height, (14d) denotes the constraint of the elevation angle, (14e) and (14f) denote the constraints of bandwidth, $B_{\max}^{u}$ and $B_{\max}^{g}$ denote the maximum bandwidth of air channel and ground channel, respectively, (14g) and (14h) denote the constraint of task split radio.

However, $P_{1}$ is a typical mixed-integer nonlinear programming (MINLP), which is challenging to deal with. The reason is following, firstly, (14b) is a integer constraint, which is difficult to solve, Secondly, $T_{k}^{g}$ and $T_{k,m}$ are not convex expression for Q and Z. Thirdly, (14d) actually is a non-affine constraint. Finally, the maximum system delay expression $\max\{T_{k}^{g},T_{k,m},\forall k,m\}$ can’t deal with directly.

## 3. Iterative Algorithm for Problem (P1)

In this section, to solve the difficult problem $P_{1}$ effectively, we first use an auxiliary variable ζ to denote the system delay, i.g., $\zeta =\max\{T_{k}^{g},T_{k,m},\forall k,m\}$, then problem $P_{1}$ can convert to $P_{2}$ as ((14a)–(14h).)

(P2):min{A,Q,Z,B,X}ζ(15a)s.t.ζ≥Tk,m,∀k,m,(15b)ζ≥Tkg,∀k,

Next we adopt an efficient iterative algorithm to solve the problem $P_{2}$ based on the SCA and BCD. Specifically, we decompose the overall MINLP into five sub-problems and convert the non-convex problem to convex problem by finding the lower bound with Taylor expansion. Then, to solve these five problems, we use some math process like standard solution method in [9], traditional optimizing tool CVX, and math analysis of closed-form.

### 3.1. UAV-TD Association Optimization

By fixing other variables {**Q**, **Z**, **B**, **X**}, we can express the UAV–TD association optimization problem as (s.t. (14a), (14b), (15a), (15b).)

(16) $$\begin{array}{cc} (P_{3}): & \min_{\left\{A\right\}}\;\zeta \end{array}$$

Because of the constraint of (14b), $P_{3}$ is a nonconvex problem with integer variable, which is hard to deal with. To make this problem can be solved easily, we relax the binary variable $a_{k,m}$ into continuous variable, which is described as

(17) $$0\leq a_{k,m}\leq 1,\;\forall k,m.$$

Then, we can rewrite the optimization problem $P_{3}$ as (s.t. (14a), (15a), (15b), (17))

(18) $$\begin{array}{cc} (P_{3.1}): & \min_{\left\{A\right\}}\;\zeta \end{array}$$

After relaxing $a_{k,m}$, we can solve $P_{3.1}$ efficiently as a typical linear programming (LP) problem, which can be solved by standard solution method [9]. We should note that $a_{k,m}$ derived by solving $P_{3.1}$ may violate the binary constraint due to relaxation. The recovery method in [21] can be used to get a strict binary solution of $a_{k,m}$.

### 3.2. UAV Horizontal Location Optimization

By fixing other variables {**A**, **Z**, **B**, **X**}, we can express UAV horizontal location optimization problem as (s.t. (14d), (15a), (15b).)

(19) $$\begin{array}{cc} (P_{4}): & \min_{\left\{Q\right\}}\;\zeta \end{array}$$

Obviously the problem $P_{4}$ is not a convex problem, because of the nonconvexity of constraints of (14d), (15a). Firstly, for the non-affine constraint (14d), we can relax it to the following form without losing the optimal solution to $P_{4}$.

(20) $$\theta_{k,m}\leq \frac{180}{\pi }\arctan\left(\frac{z_{m}}{∥q_{m}-w_{k}∥}\right).$$

It is noted that (20) is still a nonconvex constraint for $q_{m}$, but it can be convex for $∥q_{m}-w_{k}∥$. As is known to all that any convex function is globally low-bounded by its first-order Taylor expansion at any point [22]. Thus, we denote $v_{k,m}$ as

(21) $$v_{k,m}=\arctan\left(\frac{z_{m}}{∥q_{m}-w_{k}∥}\right),$$

Then, we can get the lower bound $v_{k,m}^{\mathrm{lb}}$ as

(22) $$\begin{array}{cc} v_{k,m} & \geq v_{k,m}^{\left(r\right)}-\Lambda_{k,m}^{\left(r\right)}\left(∥q_{m}-w_{k}∥-∥q_{m}^{\left(r\right)}-w_{k}∥\right)\\ \; & \overset{\Delta }{\;=}v_{k,m}^{\mathrm{lb}} \end{array}$$

where

(23a) $$\begin{array}{cc} \; & v_{k,m}^{\left(r\right)}=\arctan\left(\frac{z_{m}}{∥q_{m}^{\left(r\right)}-w_{k}∥}\right), \end{array}$$

(23b) $$\begin{array}{cc} \; & \Lambda_{k,m}^{\left(r\right)}=\frac{z_{m}}{∥q_{m}^{\left(r\right)}-w_{k}{∥}^{2}+z_{m}^{2}}. \end{array}$$

Secondly, for nonconvex constraint (15a), we relax it to

(24) $$\zeta \geq a_{k,m}\left(\frac{x_{k}l_{k}}{s_{k,m}}+x_{k}t_{k}^{u}\right),$$

where

(25) $$0\leq s_{k,m}\leq R_{k,m}.$$

Although (15a) has been relaxed, it still a nonconvex constraint for $q_{m}$. Denote $X_{k,m}^{\left(r\right)}=1+e^{\left(-\left(C_{1}+C_{2}\theta_{k,m}^{\left(r\right)}\right)\right)}$, $Y_{k,m}^{\left(r\right)}={∥q_{m}^{\left(r\right)}-w_{k}∥}^{2}+z_{m}^{2}$ and $\gamma_{k}=\frac{p\beta_{0}}{\Gamma N_{0}B_{k}^{u}}$, we can adopt the SCA method to approximate $R_{k,m}$ by its lower bound as follows using the first-order Taylor expansion,

(26) $$\begin{array}{cc} R_{k,m} & \geq R_{k,m}^{\left(r\right)}-\Omega_{k,m}^{\left(r\right)}\left(∥q_{m}-w_{k}{∥}^{2}-{∥q_{m}^{\left(r\right)}-w_{k}∥}^{2}\right)\\ \; & \;-\Xi_{k,m}^{\left(r\right)}\left(e^{\left(-\left(C_{1}+C_{2}\theta_{k,m}\right)\right)}-e^{\left(-\left(C_{1}+C_{2}\theta_{k,m}^{\left(r\right)}\right)\right)}\right)\\ \; & \overset{\Delta }{\;=}R_{k,m}^{\mathrm{lb}},\forall k,m, \end{array}$$

where the coefficients $\Omega_{k,m}^{\left(r\right)}$ and $\Xi_{k,m}^{\left(r\right)}$ are given by

(27a) $$\begin{array}{cc} \; & \Omega_{k,m}^{\left(r\right)}=B_{k}^{u}(C_{3}+\frac{C_{4}}{X_{k,m}^{\left(r\right)}})\frac{\gamma_{k}\alpha_{L}/2}{Y_{k,m}^{\left(r\right)}({\left(Y_{k,m}^{\left(r\right)}\right)}^{\alpha_{L}/2}+\gamma_{k})} \end{array}$$

(27b) $$\begin{array}{cc} \; & \Xi_{k,m}^{\left(r\right)}=B_{k}^{u}\frac{C_{4}\left(\log_{2}\left(e\right)\right)}{{\left(X_{k,m}^{\left(r\right)}\right)}^{2}}\ln\left(1+\frac{\gamma_{k}}{{\left(Y_{k,m}^{\left(r\right)}\right)}^{\alpha_{L}/2}}\right) \end{array}$$

The proof will be demonstrated in Appendix A.

Then, we can rewrite the optimization problem $P_{4}$ as ((15b), (24).)

(28a)(28b)(28c)

We can see that $P_{4.1}$ is convex now for varaible Q. Thus, by the SCA method, we get the solution of the approximation problem $P_{4.1}$ which can be solved by CVX.

### 3.3. UAV Vertical Location Optimization

By fixing other variables {**A**, **Q**, **B**, **X**}, we can express UAV vertical location optimization problem as (s.t. (14c), (14d), (15a), (15b))

(29) $$\begin{array}{cc} \left(P_{5}\right):\min_{\left\{Z\right\}} & \;\zeta \end{array}$$

The problem $P_{5}$ is not a convex problem for Z, because the nonconvexity of constraints of (14d) and (15a). Firstly, we relax non-affine constraints (14d) to (20). It is notable that the arctan function is a concave function for Z, but we still need to approximate $v_{k,m}$ by its upper bound due to the lack of support for the function of arctan in CVX, which is described as

(30) $$\begin{array}{cc} v_{k,m} & \leq v_{k,m}^{\left(r\right)}+\Psi_{k,m}^{\left(r\right)}\left(z_{m}-z_{m}^{\left(r\right)}\right)\\ \; & \overset{\Delta }{\;=}v_{k,m}^{\mathrm{up}} \end{array}$$

where

(31) $$\Psi_{k,m}^{\left(r\right)}=\frac{∥q_{m}-w_{k}∥}{∥q_{m}-w_{k}{∥}^{2}+{\left(z_{m}^{\left(r\right)}\right)}^{2}}$$

Secondly, for (15a) we must similarly convert by the first-order Taylor expansion for $R_{k,m}$ to get its low bound $R_{k,m}^{\mathrm{ld}}$ like (26), thus the process is omitted here. Then, we can rewrite the optimization problem $P_{5}$ as ( (14c), (15b) and (24).)

(32a)(32b)(32c)

We can see that $P_{5.1}$ is convex now for Z, which can be solved by CVX.

### 3.4. Bandwidth Allocation Optimization

By fixing other variables {**A**, **Q**, **H**, **X**}, we can express the bandwidth allocation optimization problem as $P_{6}$ (s.t. (14e), (14f), (15a), (15b))

(33) $$\begin{array}{cc} \; & \left(P_{6}\right):\min_{\left\{B\right\}}\;\zeta \end{array}$$

$P_{6}$ is convex optimization problem originally, which can be solved directly by CVX.

### 3.5. Task Split Radio Optimization

By fixing other variables {**A**, **Q**, **H**, **B**}, we can express the task split radio optimization problem as $P_{7}$ ((14g), (14h))

(34a)(34b)

It is worth noting that the task split radio optimization aims to balance the delay of air channel and ground channel only for some ITDs. Thus the optimal task split is obtained by $x_{k}\left(\frac{l_{k}}{{\bar{R}}_{k}}+t_{k}^{u}\right)$ = $\left(1-x_{k}\right)\left(\frac{l_{k}}{r_{k}^{g}}+t_{k}^{g}\right)$, where ${\bar{R}}_{k}$ is the offloading rate of the actual selective air channel after UAV–TD association optimization. Specifically, the overall optimal task split is given by

(35) $$x_{k}^{*}=\left\{\begin{array}{c} 0,\;\forall k\in N,\sum_{m=1}^{M}a_{k,m}=0,\\ \frac{1/r_{k}^{g}+c_{k}/f^{g}}{1/r_{k}^{g}+c_{k}/f^{g}+1/{\bar{R}}_{k}+c_{k}/f^{u}},\forall k\in N,\sum_{m=1}^{M}a_{k,m}=1,\\ 1,\;\forall k\in K,k\notin N \end{array}\right.$$

### 3.6. Convergence and Complexity Analysis

The overall proposed algorithm is displayed in Algorithm 1. First, we set the initial value of each variable. Then we get the optimization solution of each variable iteratively with fixing other variables. Then we obtain the $\zeta^{\left(r\right)}$ in the *r*-th round and update *r* to r+1 begin next iteration. Denote η as the preconfigured tolerance parameter. Until the $\frac{\zeta^{\left(r\right)}-\zeta^{\left(r+1\right)}}{\zeta^{\left(r\right)}}\leq \eta$, the algorithm is over. For a simple analysis, from above iterative algorithm, we can get

(36) $$\begin{array}{cc} \; & \zeta (A^{\left(r\right)},Q^{\left(r\right)},Z^{\left(r\right)},B^{\left(r\right)},X^{\left(r\right)})\\ \geq & \zeta (A^{\left(r+1\right)},Q^{\left(r\right)},Z^{\left(r\right)},B^{\left(r\right)},X^{\left(r\right)})\\ \geq & \zeta (A^{\left(r+1\right)},Q^{\left(r+1\right)},Z^{\left(r\right)},B^{\left(r\right)},X^{\left(r\right)})\\ \geq & \zeta (A^{\left(r+1\right)},Q^{\left(r+1\right)},Z^{\left(r+1\right)},B^{\left(r\right)},X^{\left(r\right)})\\ \geq & \zeta (A^{\left(r+1\right)},Q^{\left(r+1\right)},Z^{\left(r+1\right)},B^{\left(r+1\right)},X^{\left(r\right)})\\ \geq & \zeta (A^{\left(r+1\right)},Q^{\left(r+1\right)},Z^{\left(r+1\right)},B^{\left(r+1\right)},X^{\left(r+1\right)}) \end{array}$$

Meanwhile, it is easy to discern that a definite lower bound is available. Thus, the convergence of Algorithm 1 is guaranteed.

The complexity of Algorithm 1 is mainly dedicated by updating of the five variables, A, Q, Z, B, and X in five subproblems [22,23,24,25]. The computational complexity of UAV horizontal location and UAV vertical location are computed as $O\left({\left(KM\right)}^{3.5}\log\frac{1}{\eta }\right)$ [26], where η represents the iterative accuracy [27].Then, UAV-TD association, task split ratio and bandwidth allocation problem are liner optimization originally, where the complexity can be omitted. Thus, the overall complexity of Algorithm 1 is given as $O\left({\left(KM\right)}^{3.5}\log\frac{1}{\eta }\right)$.
**Algorithm 1:** Iterative algorithm for problem $P_{1}$1: **Initialization**: Set the UAVs’ initial horizontal location $Q^{\left(0\right)}$, vertical location $Z^{\left(0\right)}$,task split ratio $X^{\left(0\right)}$,
bandwidth allocation $B^{\left(0\right)}$; Let iteration index *r*=0.2: **repeat**  3: Solve $P_{3}$ with given $\left\{Q^{\left(r\right)},Z^{\left(r\right)},B^{\left(r\right)},X^{\left(r\right)}\right\}$, use standard solution method [9]      to find the optimal solution as $A^{\left(r+1\right)}$.  4: Solve $P_{4}$ with known $\{A^{\left(r+1\right)},Z^{\left(r\right)},B^{\left(r\right)},$ $X^{\left(r\right)}\}$, use CVX to find the optimal      solution as $Q^{\left(r+1\right)}$.  5: Solve $P_{5}$ with known $\{A^{\left(r+1\right)},Q^{\left(r+1\right)},B^{\left(r\right)},$ $X^{\left(r\right)}\}$, use CVX to find the optimal      solution as $Z^{\left(r+1\right)}$.  6: Solve $P_{6}$ with known $\{A^{\left(r+1\right)},Q^{\left(r+1\right)},Z^{\left(r+1\right)},$ $X^{\left(r\right)}\}$, use CVX to find the optimal      solution as $B^{\left(r+1\right)}$.  7: Solve $P_{7}$ with known $\{A^{\left(r+1\right)},Q^{\left(r+1\right)},Z^{\left(r+1\right)},$ $B^{\left(r+1\right)}\}$, get closed-form solution of      $X^{\left(r+1\right)}$.8: **Update** r=r+19: **Until**: $\frac{\zeta^{\left(r\right)}-\zeta^{\left(r+1\right)}}{\zeta^{\left(r\right)}}\leq \eta$. The Algorithm is over if the
fractional decrease of    the objective value is below a
threshold.

## 4. Numerical Results

In this section, numerical results are displayed to evaluate the performance of the proposed algorithm. Consider a MEC system with three GAPs, *M* = 3 UAVs and *K* = 50 TDs, where the numbers of ITD is 20 and the numbers of OTD is 30. TDs are randomly and uniformly distributed within a 2D interest area of 400×400 m${}^{2}$ and GAPs are located at the edge of area. Assume that the size of task data of each TD is same as $\{l_{k}$ = $1.6\times 10^{6}bit,\forall k\}$. The number of CPU cycles required to deal with 1-bit of task data $\{c_{k}$ = 100,∀k}. The computing resource of UAV and GAP distribute to each computing task is $f^{u}$ = $5\times 10^{8}$ Hz and $f^{g}$ = $5\times 10^{9}$ Hz, respectively. The specific environment parameters about the probabilistic LoS channel are $C_{1}$ = −0.4568, $C_{2}$ = 0.047, $C_{3}$ = −0.63, $C_{4}$ = 1.63, respectively. The initial horizontal location of UAVs are around the center of area (200,200). The initial vertical location of each UAV is $\left\{z_{m}^{\left(0\right)}=70,\forall m\right\}$. Other system parameters are listed in Table 1.

**Figure 3:**
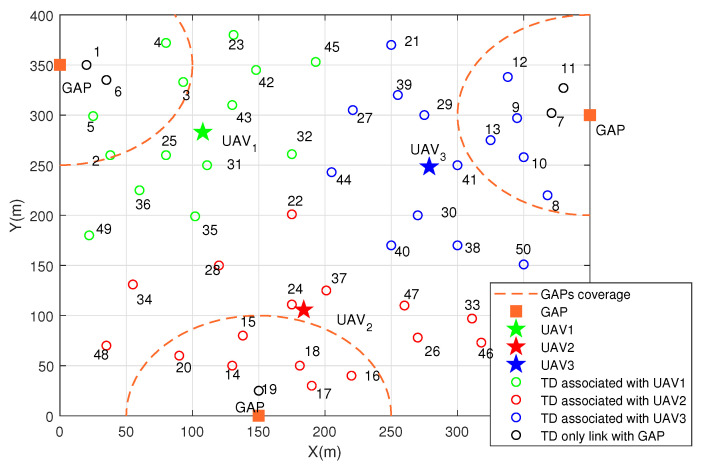
2D deployment of UAVs and association.

The 2D optimized deployment of UAVs and specific association between TDs and UAVs are shown in Figure 3, where circle represents TDs, pentagram represents UAVs, square represents GAPs, dotted line represents GAP’s coverage. It is found that three UAVs are uniformly distributed among TDs. The corresponding color between TDs and UAVs denotes the association (e.g., green TDs are associated by the green UAV). It is worth noting that there are some TDs don’t have the corresponding color with UAV, which means they aren’t associated with any UAV and complete the computing task only by GAP. That is because their locations are very close to GAP so that the offloading rate is large enough.

**Figure 4:**
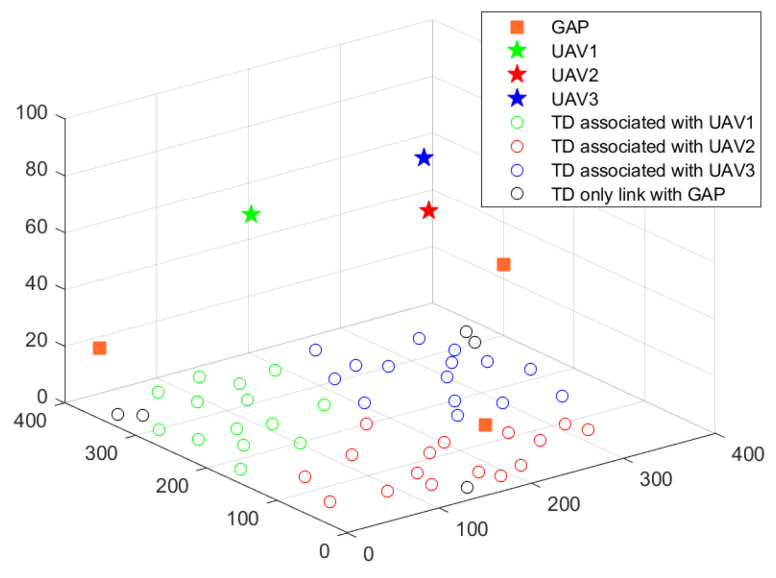
3D deployment of UAVs and association.

Figure 4 shows the 3D optimized deployment of UAVs and the association between TDs and UAVs. The vertical location of UAVs are 70.1 m, 79.5 m, 73 m, respectively. They all keep in medium rather than maximum or minimum height. That is because, for the probabilistic LoS channel, a low fly height will cause a small probability of LoS state. However, the high fly height will cause a serious path loss. Thus, the medium height finally achieves a better offloading link.

**Figure 5:**
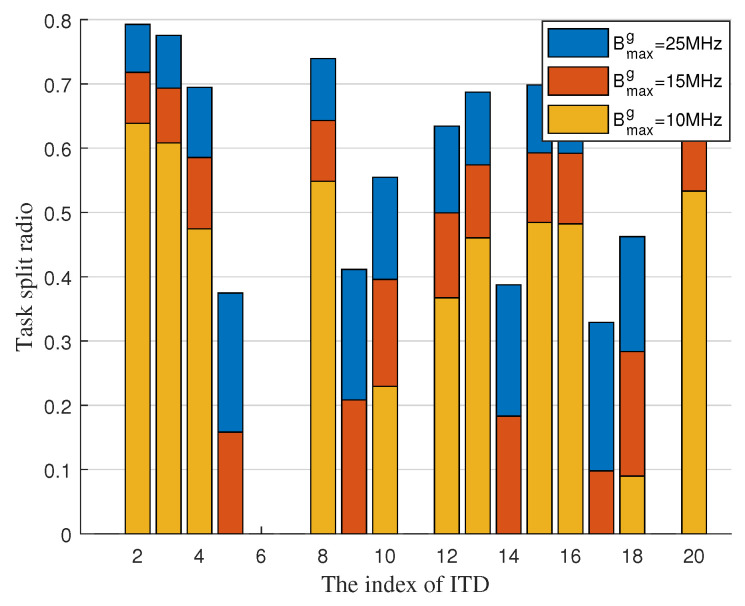
Task split radio of each ITD with maximum bandwidth of air channel.

Figure 5 displays the task split radio of each ITD under different maximum bandwidth of air channel. It is found that the task split radio of each ITD increases as maximum bandwidth of UAVs increases. That is because, more available bandwidth of UAVs can improve the offloading rate of air channel. Thus, ITD prefers to offload more task to UAV to reduce the task completion delay. Compared with each ITD, we can obtain that the task split radio of ITD is according to the distance to its associated GAP generally. The ITD which far from its associated GAP offloads more task to UAV and those close to GAP offloads less task to UAV.

**Figure 6:**
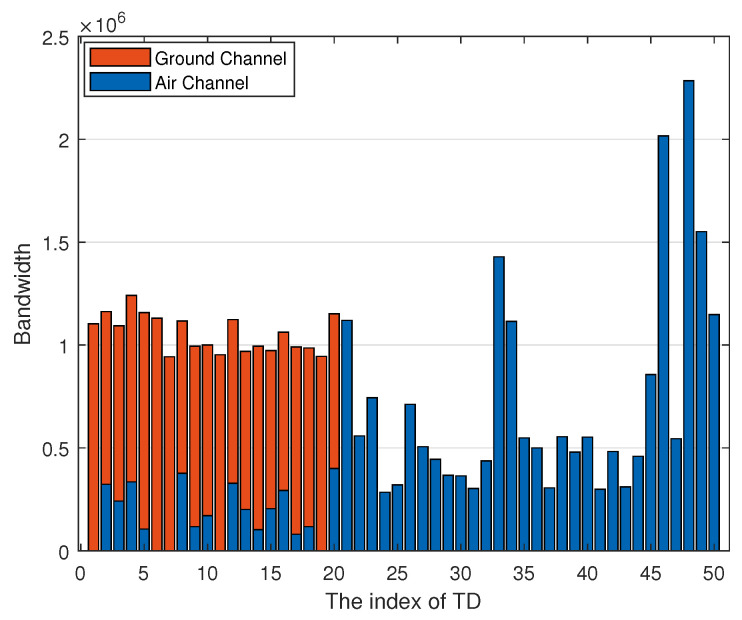
Bandwidth allocation of each TD.

Figure 6 shows the bandwidth allocation of each TD, where the blue bar denotes the bandwidth of air channel and orange bar denotes the bandwidth of ground channel. Combined with Figure 5, we can know that some ITDs such as ITD${}_{2}$, ITD${}_{3}$, and ITD${}_{4}$ mainly offload tasks by air channel but the air channel only occupies a small portion of bandwidth compared with the ground channel, which causes the air channel to have better offloading capacity then the ground channel. Meanwhile, we can obtain that the bandwidth mainly allocated to OTDs, especially for the OTDs far away from UAVs such as TD${}_{46}$, TD${}_{48}$, and TD${}_{49}$. This is because the OTD only can complete tasks via the air channel thus occupying more bandwidth.

**Figure 7:**
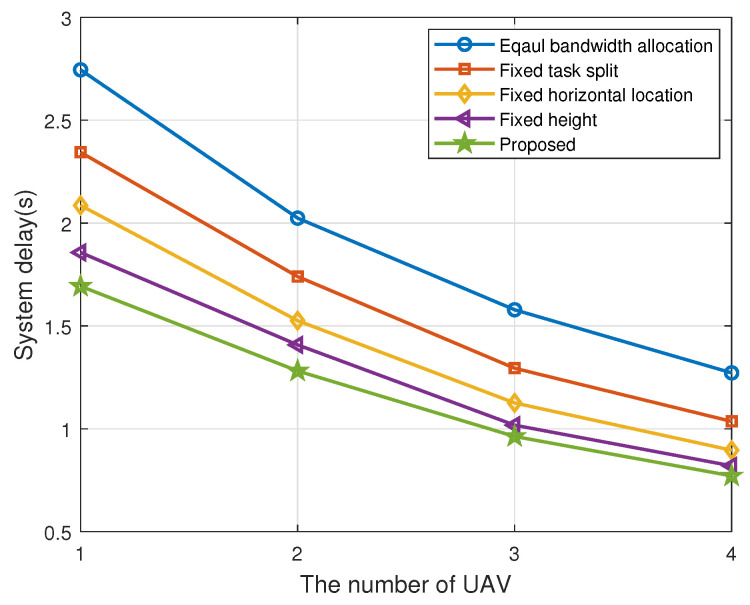
System delay with different number of UAVs.

**Figure 8:**
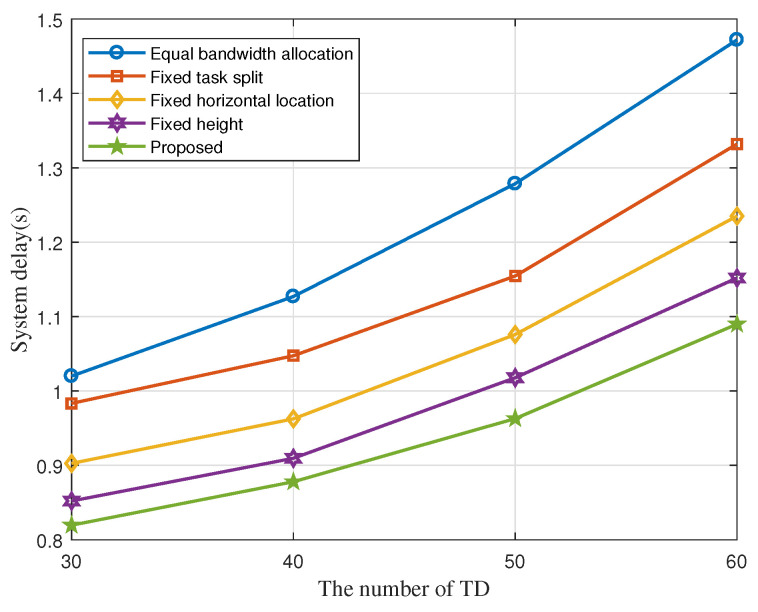
System delay with different number of TDs.

To validate the performance of our proposed algorithm, we compare it with four other benchmarks in Figure 7 and Figure 8; They are, without bandwidth allocation optimization, i.g., the bandwidth of each TD is equally divided; without task split radio optimization, i.g., task split radio of each ITD is fixed as $x_{k}$ = 0.6; without vertical location optimization, i.g., the fly height of each UAV is fixed at 70m; without horizontal location optimization, i.g., the horizontal location of UAVs are obtained by the method in [13]. It is dividing the clusters of TDs by using K-means method and then setting the centroid of the clusters as the horizontal location of UAVs. We can obtain that the system delay of all the algorithms decreases as the number of UAVs increases in Figure 7. Figure 8 shows system delay under different number of TDs. Meanwhile, the delay of our proposed algorithm is always kept at a minimum, which validates its better performance.

**Figure 9:**
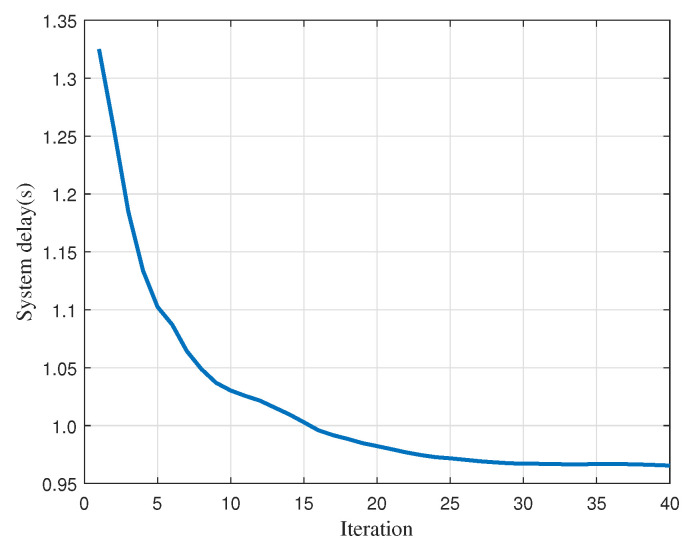
Convergence of proposed algorithm.

Finally, we show our proposed algorithm convergence versus iteration in Figure 9. It is found that the system delay decreases with iteration and the algorithm can converge in about 30 iterations. The quick convergence speed guarantees that our algorithm can be executed in a short time, and thus is feasible in a practical situation.

## 5. Discussion

Unlike in previous works, our paper presents significant novelty for the following reasons: (1) Channel model: most previous works adopt simple assumption of channel like LoS and Richan channel. To make results more accurate, we adopt a new probabilistic channel [20], which is obtained by simulation and data regression methods. (2) Offloading strategy: previous works mainly adopt a binary offloading strategy. Some works adopt a more advanced partial offloading strategy, where a TD can offload tasks to GAP and UAV servers via task splitting. We consider a more comprehensive scenario: According to the coverage of GAP, the OTD adopts a binary offloading strategy and the ITD adopts a partial offloading strategy.

Correspondingly, we formulate a novel UAV-assisted MEC system with air–ground cooperation, where UAVs and GAPs undertake computing tasks cooperatively. All the TDs get computing service simultaneously and continuously. Thanks to high mobility and flexibility of UAVs with a MEC server, the performance of the MEC system can be improved. Specifically, we aim to consistently minimize the maximum delay among TDs. To this end, we proposed an iterative algorithm by jointly optimizing UAV–TD association, UAV horizontal location, UAV vertical location, bandwidth allocation, and task split ratio. However, the overall problem is a MINLP, which is hard to directly deal with.

Compared with other works, we propose a different and effective algorithm by SCA and BCD to decompose the overall problem into five sub-problems. It is generally difficult to find the optimal solution for a nonconvex problem. Thus, SCA provides an approximate and feasible solution of nonconvex problem, which is less complex and has higher convergence speed. BCD is a traditional method to solve multivariable optimization problems. It decomposes an overall complex problem into separate sub-problems and then solves each sub-problem alternately and individually. Specifically, UAV–TD association problem is a nonconvex problem with integer variables, and we use a standard solution method in [9] to solve it. UAV horizontal location and UAV vertical location problem is also nonconvex problem, we propose an approximate solution, where the non-convex problem is converted to a convex problem by finding the lower bound using Taylor expansion. And then we solve them by the traditional optimization tool CVX. For task split radio problem, we use math analysis to get the closed-form solution, which can greatly reduce the algorithm complexity.

From the results, we can obtain that our novel MEC system with air–ground cooperation is meaningful. First, it is obvious that the high mobility of UAVs strengthens the coverage of the MEC system compared with the ground-only MEC systems of previous works. Second, with the location of TDs and GAPs known in advance, the results have shown many significant and instructive parameters regarding MEC systems in detail, including: the association between UAVs and TDs, the deployment of UAVs (horizontal location and vertical location), bandwidth allocation of each TD, which can guide the MEC system design in practice. Finally, the results have shown the effectiveness of our proposed algorithm compared with other benchmark schemes, which proves each optimization variable is meaningful and important. The results of our novel MEC system can be applied in many scenarios in real life such as for multiple devices in a wide area requiring facial recognition or automatic navigation simultaneously and continuously with a low delay.

There are still many interesting research directions that can be executed in our future work by expanding the results of this paper. Such as (i) The device-to-device (D2D) link of the OTD; (ii) The energy consumptions of TD and UAV; (iii) Multi-antennas technique of GAP and UAV; (iv)Reconfigurable intelligence surface (RIS) technique.

(i) D2D technique: D2D link is a direct communication mode between different devices. In our system model, ITD can offload the task to GAP and UAV server by task split radio but OTD can only offload the task to the UAVs. However, the computational resource offered by UAVs is limited, which may not be sufficient for the OTD for the delay-sensitive tasks. By D2D link, OTD can offload the task to nearby TD. Thus, OTD have more offloading choice and pressure of UAV is reduced.

(ii) The energy consumption of TDs and UAVs: In our system model, we aim to minimize the maximum delay among TDs in a novel multi-UAV assisted MEC system. We don’t consider the energy consumption of TDs and UAVs, which can also be feasible in some scenarios which pursue an extremely low delay regardless of energy consumption cost, such as the works [9,10,18] in our paper. However, there is some research which focuses on the endurance of both TDs and UAVs, which investigates the minimization of the weighted sum of transmission and hovering energy consumption. Thus, to improve our system model applicability, minimizing delay with the constraint of the energy consumption of UAVs and TDs will be important in future work.

(iii) Multi-antenna technique: We assume all the communication nodes (TD, GAP, and UAV) are equipped with a single antenna. Because we mainly focus on the analysis of communication link delay. Single antennae will simplify the analysis process. However a multi-antenna technique has better performance in terms of energy consumption, signal transmission and reception, and improving the channel capacity. Some related work like [15] has adopted a multi-antenna technique in UAV-assisted MEC systems, such a scenario is worthy of investigation in future work [28].

(iv) RIS technique: RIS is a two-dimensional surface composed of a large number of passive units arranged mostly in subwavelength inter-element spacing with the property of manipulating the electromagnetic waves, such as scattering, reflection, and absorption. It can intelligently reconfigure the wireless communication environment, so as to significantly improve the performance of wireless communication networks. The combination of RIS and UAV-assisted MEC system can improve the quality of the communication channel and strengthens the service coverage.

## 6. Conclusions

In this paper, we studied a novel framework of MEC networks with air–ground cooperation, where several GAPs and UAVs offer service for TDs cooperatively. Considering TD fairness, a joint optimization problem of UAV–TD association, UAV horizontal location, UAV vertical location, bandwidth allocation, and task split ratio was formulated. In order to solve this MINLP, we proposed an alternating iterative algorithm based on successive convex approximation and the block coordinate descent methods. Finally, the simulation results validated the effectiveness of the proposed algorithm.

## Figures and Tables

**Table 1:** Parameter setting in simulation.

Description	Parameter	Value
Altitude of GAPs	$H_{g}$	20 m
Transmit power of TDs	*p*	0.1 w
Minimum Height of UAV	$H_{\min}$	50 m
Maximum Height of UAV	$H_{\max}$	100 m
Noise power spectral density	$N_{0}$	−169 dBm
Reference channel power	$\beta_{0}$	−60 dB
SNR gap	Γ	8.2 dB
Accuracy tolerance	η	$10^{-4}$
Path loss exponent(LoS)	$\alpha_{L}$	2.5
Path loss exponent(Rayleigh)	$\alpha_{R}$	3.5
Total bandwidth for UAVs	$B_{\max}^{u}$	25 MHz
Total bandwidth for each GAP	$B_{\max}^{g}$	6 MHz

## Data Availability

Data available on request.

## Appendix A

For any convex function g(x,y), we adopt first-order Taylor expansion with the known point $x_{0}$, $y_{0}$, then we can get

(A1) $$\begin{array}{cc} g(x,y)\geq & g\left(x_{0},y_{0}\right)+g_{x}^{{}^{\prime }}\left(x_{0},y_{0}\right)\left(x-x_{0}\right)\\ \; & +g_{y}^{{}^{\prime }}\left(x_{0},y_{0}\right)\left(y-y_{0}\right) \end{array}$$

Then, we set g(x,y) = $\left(C_{3}+\frac{C_{4}}{x}\right)\log_{2}\left(1+\frac{\gamma }{y}\right)$, which is an obviously convex function when x>0, y>0 and constant γ > 0, $C_{3}$ > 0, $C_{4}$ > 0. Thus, we can obtain the form of low bound as (26).
